# An epidemiological study to assess *Plasmodium falciparum* parasite prevalence and malaria control measures in Burkina Faso and Senegal

**DOI:** 10.1186/s12936-017-1715-1

**Published:** 2017-02-06

**Authors:** Aldiouma Diallo, Ali Sié, Sodiomon Sirima, Khadime Sylla, Mahmadou Ndiaye, Mamadou Bountogo, Espérance Ouedraogo, Roger Tine, Assane Ndiaye, Boubacar Coulibaly, Alphonse Ouedraogo, Babacar Faye, El Hadji Ba, Guillaume Compaore, Alfred Tiono, Cheikh Sokhna, Maurice Yé, Amidou Diarra, Edith Roset Bahmanyar, Melanie De Boer, Jean-Yves Pirçon, Effua Abigail Usuf

**Affiliations:** 1 Centre de Recherche de Niakhar, Institut de Recherche pour le Développement, Dakar, Senegal; 2 Centre de Recherche en Santé de Nouna, Nouna, Burkina Faso; 3 Centre National de Recherche et de Formation sur le Paludisme (CNRFP), Ouagadougou, Burkina Faso; 4 Centre de Recherche de Koer Socé, Service de Parasitologie Médicale, Faculté de Médecine, Université Cheikh Anta Diop, Dakar, Senegal; 5 Profa Foundation, Vaud, Switzerland; 6 GSK Vaccines, Wavre, Belgium

**Keywords:** Malaria, Burkina Faso, Senegal, Epidemiological study, Parasite prevalence, Risk factors, Preventive interventions

## Abstract

**Background:**

Malariometric information is needed to decide how to introduce malaria vaccines and evaluate their impact in sub-Saharan African countries.

**Methods:**

This cross-sectional study (NCT01954264) was conducted between October and November, 2013, corresponding to the high malaria transmission season, in four sites with Health and Demographic Surveillance Systems (DSS) [two sites with moderate-to-high malaria endemicity in Burkina Faso (Nouna and Saponé) and two sites with low malaria endemicity in Senegal (Keur Socé and Niakhar)]. Children (N = 2421) were randomly selected from the DSS lists of the study sites and were stratified into two age groups (6 months–4 years and 5–9 years). A blood sample was collected from each child to evaluate parasite prevalence of *Plasmodium falciparum* and other *Plasmodium* species and gametocyte density by microscopy, and rapid diagnosis test in the event of fever within 24 h. Case report forms were used to evaluate malaria control measures and other factors.

**Results:**

*Plasmodium falciparum* was identified in 707 (29.2%) children, with a higher prevalence in Burkina Faso than Senegal (57.5 vs 0.9% of children). In Burkina Faso, prevalence was 57.7% in Nouna and 41.9% in Saponé in the 6 months–4 years age group, and 75.4% in Nouna and 70.1% in Saponé in the 5–9 years age group. Infections with other *Plasmodium* species were rare and only detected in Burkina Faso. While mosquito nets were used by 88.6–97.0 and 64.7–80.2% of children in Burkina Faso and Senegal, other malaria control measures evaluated at individual level were uncommon. In Burkina Faso, exploratory analyses suggested that use of malaria treatment or any other medication within 14 days, and use of insecticide spray within 7 days decreased the prevalence of malaria infection; older age, rural residence, natural floor, grass/palm roof, and unavailability of electricity in the house were factors associated with increased malaria occurrence.

**Conclusions:**

*Plasmodium falciparum* infection prevalence in children younger than 10 years was 57.5% in Burkina Faso and 0.9% in Senegal, and variability was observed, among others, by age, study site and malaria control measures.

## Background

In sub-Saharan Africa, malaria remains a major cause of morbidity and mortality, especially in young children [1]. In 2013, when the current study was conducted, approximately 198 million malaria cases occurred globally, causing 584,000 deaths. Most cases (90%) occurred in Africa, and most deaths (78%) were in children under 5 years of age [2].

In many African countries, malaria control programmes have been implemented since 2000, including the use of insecticide-treated nets (ITNs), long-lasting insecticidal nets (LLINs), indoor residual spraying of insecticides (IRS), rapid diagnostic tests (RDTs), and effective anti-malaria medicines [1]. Although these interventions have been associated with a reduction in malaria incidence rates, the increasing problem of multi-drug resistance and insecticide resistance highlights the need for new tools, especially in areas of moderate-to-high malaria transmission intensity (MTI) [1, 3–7].

Adding a malaria vaccine to the control programmes has been identified as a key component to complement current interventions [8, 9]. RTS,S/AS01 is the first malaria vaccine that underwent large-scale phase 3 evaluation in Africa [4, 10–12] and received a positive regulatory assessment by the European Medicines Agency in July 2015 [13]. Before considering its introduction for routine use, the World Health Organization (WHO) recommended further evaluation of its four-dose vaccination schedule in a series of pilot implementations in moderate-to-high transmission settings in sub-Saharan Africa [1].

Malariometric information is needed to guide decisions on how to prioritize interventions and introduce malaria vaccines. Among others, the level of malaria transmission, which is very heterogeneous within geographical areas, may have an effect on the efficacy of malaria control measures, including RTS,S/AS01 [12, 14]. The objectives of the present study were to evaluate the parasite prevalence (PP): an indirect, cost-effective way to evaluate the force of malaria transmission) of *Plasmodium falciparum* and other *Plasmodium* species, the extent of use of malaria control measures, and other factors in four sites, where phase IV studies on RTS,S/AS01 were expected to be conducted. These results are needed to anticipate the impact of RTS,S/AS01 if this vaccine is implemented in focalized pilot programmes or national vaccination programmes.

## Methods

### Study design and area

This epidemiological, cross-sectional study was carried out at four sites with Health and Demographic Surveillance Systems (HDSS) in Burkina Faso and Senegal (NCT01954264). The first site was located in the area of Nouna at about 300 km from Ouagadougou (northwestern Burkina Faso), where malaria is hyper- to holo-endemic, the malaria transmission peak occurs at the end of the rainy season (June to October), and reduced transmission is observed during the dry season (December to May) [15]. The second site in Burkina Faso was located in Saponé (30 km southwest of Ouagadougou), where malaria transmission is also seasonal, and the peak is observed at the end of the rainy season in September [16]. The third site was located in the Keur Socé area (200 km southeast of Dakar, Senegal), which is characterized by the alternation of a long dry season and a short rainy season from July to November, and has a peak in malaria transmission between October and November [17, 18]. The last site was located in the Niakhar area (115 km southeast of Dakar, Senegal), where malaria is meso-endemic and transmission is seasonal from September to December [19].

### Study population

Participants were children aged ≥6 months to <10 years, whose parents or legally acceptable representative had provided informed consent prior to any study-specific procedure. Children in care, or actively participating in any trial involving administration of an investigational malaria vaccine or drug were excluded. Children were randomly selected from the HDSS list at each site, and were stratified into two age groups for the analyses, as defined by the Joint Technical Expert Group (JTEG) criteria: children aged 6 months–4 years and children aged 5–9 years. Analyses were performed on all evaluable children for whom at least one laboratory result was available.

### Data collection

**Fig. 1:**
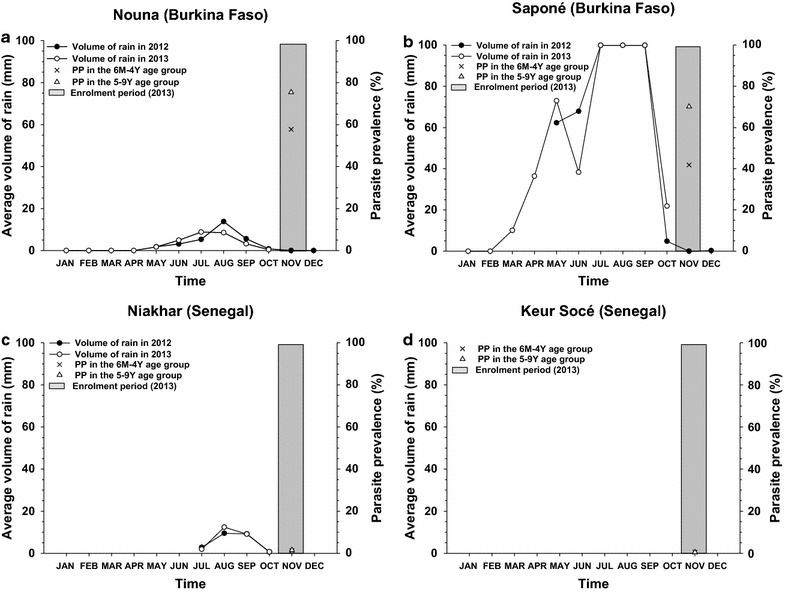
*Plasmodium falciparum* parasite prevalence, annual rainfall and enrolment period for the study center of **a** Nouna (Burkina Faso), **b** Saponé (Burkina Faso), **c** Niakhar (Senegal), and **d** Keur Socé (Senegal). *PP* parasite prevalence, *M* months, *Y* years

This study was conducted during or just after the rainy season, at the peak of malaria transmission. Data were collected at one visit between 19 October and 25 November, 2013, and no follow-up was done (Fig. 1). Internet-based electronic case report forms were used to record demographic details, relevant medical history, malaria control measures used in the household, malaria risk factors, anti-malaria medication and other medication received within 14 days, and history of fever in the last 24 h. Axillary body temperature at the time of the survey was measured by trained personnel according to the study protocol.

The malaria control measures evaluated at the individual level were the use of mosquito bed nets the night before the visit, mosquito coils, insecticide sprays, and commercial and traditional repellents over 7 days, and IRS on interior walls in previous 12 months. The other factors evaluated in this study were age, gender, study centre, number of persons living in the same house, localization (rural vs urban area; town vs countryside), main house construction material (walls, floor, roof, windows/eaves, nets), main source of drinking water, and availability of electricity.

Each study site was requested to provide centre-specific information about control measures from the malaria control programme in the study area and, if available, meteorological data for the study site, such as rainfall and temperature. The information was collected in the form of a questionnaire recorded in a separate database.

### Biological methods

Approximately 200 µL of whole blood was collected from all participants. A RDT was performed in the event of recent history of fever (axillary temperature ≥37.5 °C measured at the time of visit or reported in the previous 24 h). The RDT was used to detect HRP-II (Histidine-rich protein II) specific to *P. falciparum* and pLDH (*Plasmodium* lactate dehydrogenase) specific to *Plasmodium vivax* (SD BIOLINE Malaria Ag P.f/P.v test). If the RDT was positive or the child was identified as being parasite-positive following microscopy, treatment was given according to national guidelines.

The presence of parasites on capillary blood samples was assessed by 100-field microscopic examination, assuming 8000 leukocytes/µl of blood. The count was made by species *(P. falciparum, P. malariae, P. vivax,* or *Plasmodium ovale*), and counts for *P. falciparum* were made for both gametocytes and asexual parasites. Two slides, each containing a thick and thin blood smear, were prepared; positive parasitaemia was identified on the thick blood film, and species on the thin blood film, except in case of low parasitaemia. The parasite presence and density were determined independently by two readers for the same slide; if readings were judged to be discordant, a third independent read was organised. A child was defined as infected by a specific parasite if at least two readings were positive for the corresponding parasite. If the final decision was positive, the parasite density (parasites/μL) was calculated as the geometric mean of the two positive readings (two geometrically closest readings in the case of three positive reads). The following classes were used to categorize the parasite densities: low (<2500 parasites/μL); medium (2500–9999 parasites/μL); high (10,000–19,999 parasites/μL), and very high (≥20,000 parasites/μL).

A child was defined as having gametocytes when gametocytes were detected during at least one reading. In children with a positive status, the gametocyte density (units/μL) was defined as the geometric mean of the readings of the positive slides.

### Statistical methods

The target sample size was 2400 children (600 at each participating site), according to the following stratification by age groups: 6 months-<1 year-olds (N = 60), 1 and 2 year-olds (N = 120 per group), 3 and 4 year-olds (N = 50 per group), and 5, 6, 7, 8, and 9 year-olds (N = 40 per group). To allow for 10% non-response, 660 children over all age categories were randomly selected from the demographic surveillance system databases of each site. This sample size ensured sufficiently narrow confidence intervals (CIs) around centre-wise PP estimates (with a maximum relative standard error of 0.25) in children from the 6 months–4 years (JTEG definition) and 29 years (WHO definition) age groups for sites with low (<10%), moderate (10–50%), or high (>50%) endemicity.

Continuous variables were described with mean, standard deviation (SD), median, and range. Categorical variables were described in frequency tables with absolute numbers and percentages. For some categorical variables, 95% CIs were also calculated.

Potential differences between groups were detected in exploratory analyses based on non-overlapping 95% CIs. The impact of various malaria control measures and other factors on the *P. falciparum* infection status was evaluated as exploratory analysis by centre in univariate logistic regression analyses and summarized using unadjusted odds ratios (ORs) with 95% CIs. A potential impact was detected if the 95% CI of the unadjusted ORs did not include the value ‘1’. The statistical analyses were performed using the Statistical Analysis Systems, version 9.2.

## Results

### Characteristics of the study participants

In this study, 2421 children were enrolled, with a similar distribution among the four sites. A few minor protocol deviations were reported (inconsistencies in terms of RDT testing based on the temperature), but no child was excluded from the total cohort randomly selected to participate in the study. A total of 1210 children were enrolled in Burkina Faso (610 in Nouna and 600 in Saponé) and 1211 children in Senegal (603 in Keur Socé centre and 608 in Niakhar). Among all participants, 52.3% were boys, with an even gender distribution among the four centres. The proportion of children living in rural areas was higher in the two Senegalese centres (≥99.8%) and in Saponé (≥98.7%) than in Nouna (53.6% in the 6 months–4 years and 51.7% in the 5–9 years age groups) (Table 1).

**Table 1:** Baseline characteristics of the study participants

Site	Age classes	Characteristic	Values
Nouna (Burkina Faso)	Overall	N	610
		Age, mean years (SD)	4.04 (2.75)
		Male, n (%)	315 (51.6%)
		Female, n (%)	295 (48.4%)
	6 months–4 years	N	407
		Rural area, n (% [95% CI])	218 (53.6% [48.6, 58.5])
		Fever in the last 24 h, n (% [95% CI])	100 (24.6% [20.5, 29.1])
		Malaria treatment in past 14 days, n (% [95% CI])	90 (22.1% [18.2, 26.5])
		Other medications in past 14 days, n (% [95% CI])	177 (43.5% [38.6, 48.5])
	5–9 years	N	203
		Rural area, n (% [95% CI])	105 (51.7% [44.6, 58.8])
		Fever in the last 24 h, n (% [95% CI])	33 (16.3% [11.5, 22.1])
		Malaria treatment in past 14 days, n (% [95% CI])	34 (16.7% [11.9, 22.6])
		Other medications in past 14 days, n (% [95% CI])	66 (32.5% [26.1, 39.4])
Saponé (Burkina Faso)	Overall	N	600
		Age, mean years (SD)	4.01 (2.74)
		Male, n (%)	315 (52.5%)
		Female, n (%)	285 (47.5%)
	6 months–4 years	N	399
		Rural area, n (% [95% CI])	394 (98.7% [97.1, 99.6])
		Fever in the last 24 h, n (% [95% CI])	70 (17.5% [13.9, 21.6])
		Malaria treatment in past 14 days, n (% [95% CI])	44 (11.0% [8.1, 14.5])
		Other medications in past 14 days, n (% [95% CI])	56 (14.0% [10.8, 17.8])
	5–9 years	N	201
		Rural area, n (% [95% CI])	200 (99.5% [97.3, 100])
		Fever in the last 24 h, n (% [95% CI])	12 (6.0% [3.1, 10.2])
		Malaria treatment in past 14 days, n (% [95% CI])	10 (5.0% [2.4, 9.0])
		Other medications in past 14 days, n (% [95% CI])	12 (6.0% [3.1, 10.2])
Keur Socé (Senegal)	Overall	N	603
		Age, mean years (SD)	4.12 (2.70)
		Male, n (%)	321 (53.2%)
		Female, n (%)	282 (46.8%)
	6 months–4 years	N	399
		Rural area, n (% [95% CI])	399 (100% [99.1, 100])
		Fever in the last 24 h, n (% [95% CI])	48 (12.0% [9.0, 15.6])
		Malaria treatment in past 14 days, n (% [95% CI])	1 (0.3% [0.0, 1.4])
		Other medications in past 14 days, n (% [95% CI])	31 (7.8% [5.3, 10.8])
	5–9 years	N	204
		Rural area, n (% [95% CI])	204 (100% [98.2, 100])
		Fever in the last 24 h, n (% [95% CI])	20 (9.8% [6.1, 14.7])
		Malaria treatment in past 14 days, n (% [95% CI])	1 (0.5% [0.0, 2.7])
		Other medications in past 14 days, n (% [95% CI])	8 (3.9% [1.7, 7.6])
Niakhar (Senegal)	Overall	N	608
		Age, mean years (SD)	3.98 (2.76)
		Male, n (%)	315 (51.8%)
		Female, n (%)	293 (48.2%)
	6 months–4 years	N	409
		Rural area, n (% [95% CI])	408 (99.8% [98.6, 100])
		Fever in the last 24 h, n (% [95% CI])	37 (9.0% [6.4, 12.3])
		Malaria treatment in past 14 days, n (% [95% CI])	5 (1.2% [0.4, 2.8])
		Other medications in past 14 days, n (% [95% CI])	56 (13.7% [10.5, 17.4])
	5–9 years	N	199
		Rural area, n (% [95% CI])	199 (100% [98.2, 100])
		Fever in the last 24 h, n (% [95% CI])	11 (5.5% [2.8, 9.7])
		Malaria treatment in past 14 days, n (% [95% CI])	2 (1.0% [0.1, 3.6])
		Other medications in past 14 days, n (% [95% CI])	13 (6.5% [3.5, 10.9])

*N* total number of participants, *SD* standard deviation, *n* (%) number (percentage) of participants in a given category, *M* months, *Y* years, *95*% *CI* 95% confidence interval

Fever in the last 24 h was reported in 24.6, 17.5, 12.0, and 9.0% of children aged 6 months–4 years, and in 16.3, 6.0, 9.8, and 5.5% of children aged 5–9 years in Nouna, Saponé, Keur Socé, and Niakhar, respectively (Table 1). The fever started, on average, 3 days before the study visit. At the study visit, 7.6, 8.3, 2.3, and 13.9% of children aged 6 months–4 years and 6.9, 2.0, 2.0, and 13.6% of children aged 5–9 years had fever in Nouna, Saponé, Keur Socé, and Niakhar, respectively. Among children with fever, the mean (SD) temperature was 38.0 °C (0.6), 38.1 °C (0.7), 38.4 °C (0.8), and 37.9 °C (0.5) in Nouna, Saponé, Keur Socé, and Niakhar, respectively.

In the Senegalese centres, ≤1.2% of children received any malaria treatment in the past 14 days, while in Burkina Faso, this percentage was 22.1 and 16.7% in Nouna, and 11.0 and 5.0 in Saponé in the 6 months–4 years and 5–9 years age groups, respectively (Table 1). The average duration of malaria treatment was 3 days, and for 14.1% of these children, the malaria treatment was still ongoing at the visit. In Burkina Faso, 149 out of the 178 children who received an anti-malarial were treated with artesunate-amodiaquine (83.7%), while in Senegal, four/nine children were treated with dihydroartemisinin-piperaquine (Niakhar centre), four/nine with artemether-lumefantrine, and one/nine with an unknown medication. The proportion of children who had received other medications in the past 14 days ranged from 3.9 to 43.5% (Table 1).

### Parasite prevalence

**Fig. 2:**
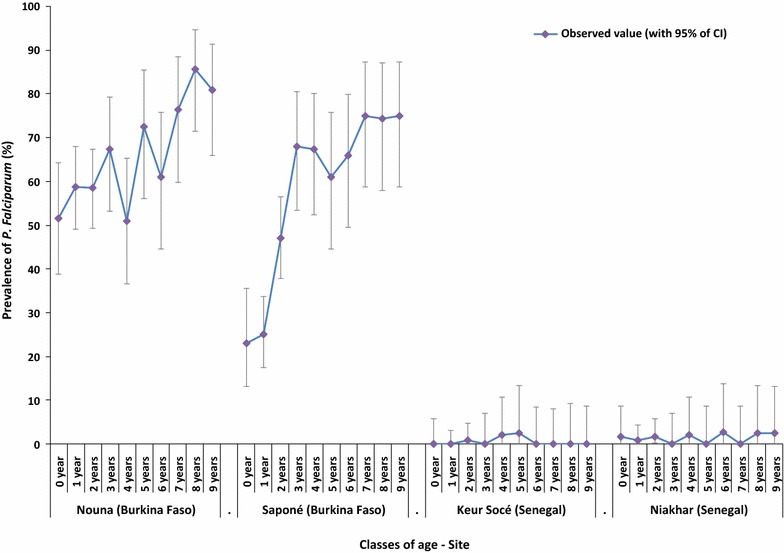
*Plasmodium falciparum* parasite prevalence by site according to age. 95% CI 95% confidence interval

Malaria infections caused by *P. falciparum* were detected in 29.2% of children; 57.9% of them were in the 6 months–4 years age group and 42.1% in the 5–9 years age group (Table 2; Fig. 1). The vast majority of infections (98.4%) were observed in Burkina Faso. In Nouna and Saponé, 57.7 and 41.9% of children in the 6 months–4 years age group, and 75.4 and 70.1% of children in the 5–9 years age group were infected, respectively. In Keur Socé, 0.5% of children were infected in both age groups, while infections were observed in 1.2% of children in the 6 months–4 years age group and 1.5% of children in the 5–9 years age group in Niakhar. In Burkina Faso, the *P. falciparum* PP seemed to increase with age during the first 9 years of life, while it seemed more uniform in Senegal (Fig. 2). Among the 404 children with results available for both RDT and microscopy, the degree of agreement between the two diagnostic tests was 85.3%.

**Table 2:** *Plasmodium falciparum* parasite prevalence by site and age group

Age group	Site	N	n	PP (95% CI)
6 months–4 years	Nouna (Burkina Faso)	407	235	57.74 (52.78, 62.59)
	Saponé (Burkina Faso)	399	167	41.85 (36.97, 46.87)
	Keur Socé (Senegal)	399	2	0.50 (0.06, 1.80)
	Niakhar (Senegal)	409	5	1.22 (0.40, 2.83)
5–9 years	Nouna (Burkina Faso)	203	153	75.37 (68.85, 81.13)
	Saponé (Burkina Faso)	201	141	70.15 (63.31, 76.38)
	Keur Socé (Senegal)	204	1	0.49 (0.01, 2.70)
	Niakhar (Senegal)	199	3	1.51 (0.31, 4.34)

*N* number of children with known results for *P. falciparum* parasitaemia test, *n* number of children infected by *P. falciparum*, *PP* parasite prevalence, *95*% *CI* 95% confidence interval, *M* months, *Y* years

In Burkina Faso, the minimum parasite density recorded was 16 parasites/µL in children aged 6 months–4 years and 11.31 parasites/µL in children aged 5–9 years. In Niakhar, similar results were obtained, with a minimum parasite density of 28.67 parasites/µL in children aged 6 months–4 years and 7.75 parasites/µL in children aged 5–9 years. In Keur Socé, however, the minimum parasite density recorded in the three children positive for *P. falciparum* was higher: 244.75 parasites/µL in the two children aged 6 months–4 years and 61,744.63 parasites/µL in the children aged 5–9 years.

In Nouna, potential differences in the proportion of children who received any malaria treatment in the past 14 days were detected between infected and non-infected children: 15.3 vs 31.4% for 6 months–4 years old children, and 10.5 vs 36.0% for 5–9 years old children. In 6 months–4 years old children in Saponé, potential differences were detected in terms of proportion of children who received any malaria treatment in the past 14 days (4.2% of infected children vs 15.9% of non-infected) and in terms of children with fever within the last 24 h (25.7% of infected children vs 11.6% of non-infected). In 5–9 years old children in Saponé, 0.7 and 15.0% of infected and non-infected children received any malaria treatment in the past 14 days, and 2.1 and 15.0% of infected and non-infected children received other medication in the past 14 days, respectively.

There was a greater likelihood of fever in the last 24 h for children with very high malaria parasite density (48.4%) compared to low (15.8%) or negative (10.8%) parasite density. Moreover, the overall proportion of children hospitalized for malaria in the last 3 months tended to be higher in children with very high malaria parasite density (6.5%) compared to those with low (3.4%) or negative parasite results (1.8%).

Gametocytes were more frequently detected in children from the centres with a higher PP (12.3 and 11.8% in Nouna, and 18.5 and 15.9% in Saponé in the 6 months–4 years and 5–9 years age groups, respectively). In the Senegalese centres, the number of gametocytes was extremely low, with a proportion of 0.3 and 0.0% in Keur Socé, and 1.0 and 0.5% in Niakhar in the same age groups. However, gametocytes were also frequently detected in children with low parasite density: 39.5% of 6 months–4 years old children and 64.9% of 5 to 9 years old children with gametocytes had a parasite density <2500 parasites/µL. *P. falciparum* was identified in 80.6 and 86.0% of children with gametocytes in the 6 months–4 years and 5–9 years age groups, respectively.

No other *Plasmodium* species were identified in the Senegalese centres. In Burkina Faso, infection with *P. malariae* was observed in eight/407 (2.0%) and 12/203 (5.9%) children in Nouna, and 15/399 (3.8%) and 20/201 (10.0%) children in Saponé in the 6 months–4 years and 5–9 years age groups, respectively. Infection with *P. ovale* was observed in nine children. No infection with *P. vivax* was recorded.

### Impact of malaria control measures and other factors

The actual implementation of recommended malaria control measures was evaluated at the site level. ITNs were distributed for free in Niakhar, but were not free of charge in Nouna. No policy regarding the distribution of ITNs was implemented in Saponé, and no information was reported for Keur Socé. LLINs were distributed for free to pregnant women in Nouna, to the entire population with a replacement period of ≥24 months in Saponé and Keur Socé, and to children younger than 5 years and pregnant women in Niakhar. Artemisinin-based combination therapy (ACT) was given for free to patients with malaria symptoms and positive RDT test at the four sites, except in Saponé where ACT was not free of charge.

Among the malaria control measures evaluated at the individual level, mosquito nets were the most frequently used in both countries (Table 3). In Burkina Faso, 88.6–97.0% of children slept under a mosquito net the night before the visit: the vast majority were new (≥95.5% less than 1 year) and impregnated (≥98.5%), and less than 5% were pierced/torn, as reported to the investigators. In Senegal, 64.7–80.2% of children slept under a mosquito net the night before the visit. In Keur Socé, ≥99.2% of bed nets were new and impregnated, but ≥41.2% of them were also pierced/torn. In Niakhar, ≤18.3% of bed nets were new and impregnated, and ≥43.0% of them were pierced/torn. In Burkina Faso, no differences in parasite density were observed between children who slept or did not sleep under a bed net the night before the visit.

**Table 3:** Malaria control measures by site, age group and *Plasmodium falciparum* infection status

Site	Age group	Malaria control measure used	Infected children N = 707		Non-infected children N = 1714		Total N = 2421	
			N	% (95% CI)	N	% (95% CI)	N	% (95% CI)
Nouna (Burkina Faso)	6 months–4 years	Mosquito net in the night before visit	220	93.6 (89.7, 96.4)	169	98.3 (95.0, 99.6)	389	95.6 (93.1, 97.4)
		Mosquito coils over 7 days	17	7.2 (4.3, 11.3)	11	6.4 (3.2, 11.2)	28	6.9 (4.6, 9.8)
		Insecticide sprays over 7 days	7	3.0 (1.2, 6.0)	14	8.1 (4.5, 13.3)	21	5.2 (3.2, 7.8)
		Commercial repellents over 7 days	1	0.4 (0.0, 2.3)	1	0.6 (0.0, 3.2)	2	0.5 (0.1, 1.8)
		Residual spray on interior walls in past 12 months	1	0.4 (0.0, 2.3)	0	0.0 (0.0, 2.1)	1	0.2 (0.0, 1.4)
	5–9 years	Mosquito net in the night before visit	149	97.4 (93.4, 99.3)	48	96.0 (86.3, 99.5)	197	97.0 (93.7, 98.9)
		Mosquito coils over 7 days	17	11.1 (6.6, 17.2)	6	12.0 (4.5, 24.3)	23	11.3 (7.3, 16.5)
		Insecticide sprays over 7 days	8	5.2 (2.3, 10.0)	4	8.0 (2.2, 19.2)	12	5.9 (3.1, 10.1)
		Commercial repellents over 7 days	2	1.3 (0.2, 4.6)	1	2.0 (0.1, 10.6)	3	1.5 (0.3, 4.3)
Saponé (Burkina Faso)	6 months–4 years	Mosquito net in the night before visit	161	96.4 (92.3, 98.7)	219	94.4 (90.6, 97.0)	380	95.2 (92.7, 97.1)
		Mosquito coils over 7 days	2	1.2 (0.1, 4.3)	1	0.4 (0.0, 2.4)	3	0.8 (0.2, 2.2)
		Commercial repellents over 7 days	1	0.6 (0.0, 3.3)	0	0.0 (0.0, 1.6)	1	0.3 (0.0, 1.4)
		Residual spray on interior walls in past 12 months	0	0.0 (0.0, 2.2)	2	0.9 (0.1, 3.1)	2	0.5 (0.1, 1.8)
	5–9 years	Mosquito net in the night before visit	125	88.7 (82.2, 93.4)	53	88.3 (77.4, 95.2)	178	88.6 (83.3, 92.6)
		Mosquito coils over 7 days	2	1.4 (0.2, 5.0)	0	0.0 (0.0, 6.0)	2	1.0 (0.1, 3.5)
		Residual spray on interior walls in past 12 months	2	1.4 (0.2, 5.0)	0	0.0 (0.0, 6.0)	2	1.0 (0.1, 3.5)
Keur Socé (Senegal)	6 months–4 years	Mosquito net in the night before visit	2	100 (15.8, 100)	275	69.3 (64.5, 73.8)	277	69.4 (64.6, 73.9)
		Mosquito coils over 7 days	0	0.0 (0.0, 84.2)	33	8.3 (5.8, 11.5)	33	8.3 (5.8, 11.4)
		Insecticide sprays over 7 days	0	0.0 (0.0, 84.2)	26	6.5 (4.3, 9.4)	26	6.5 (4.3, 9.4)
		Traditional repellents over 7 days	0	0.0 (0.0, 84.2)	31	7.8 (5.4, 10.9)	31	7.8 (5.3, 10.8)
		Residual spray on interior walls in past 12 months	0	0.0 (0.0, 84.2)	12	3.0 (1.6, 5.2)	12	3.0 (1.6, 5.2)
	5–9 years	Mosquito net in the night before visit	0	0.0 (0.0, 97.5)	132	65.0 (58.0, 71.6)	132	64.7 (57.7, 71.3)
		Mosquito coils over 7 days	1	100 (2.5, 100)	18	8.9 (5.3, 13.7)	19	9.3 (5.7, 14.2)
		Insecticide sprays over 7 days	0	0.0 (0.0, 97.5)	4	2.0 (0.5, 5.0)	4	2.0 (0.5, 4.9)
		Traditional repellents over 7 days	0	0.0 (0.0, 97.5)	20	9.9 (6.1, 14.8)	20	9.8 (6.1, 14.7)
		Residual spray on interior walls in past 12 months	0	0.0 (0.0, 97.5)	6	3.0 (1.1, 6.3)	6	2.9 (1.1, 6.3)
Niakhar (Senegal)	6 months–4 years	Mosquito net in the night before visit	5	100 (47.8, 100)	323	80.0 (75.7, 83.7)	328	80.2 (76.0, 83.9)
		Mosquito coils over 7 days	0	0.0 (0.0, 52.2)	11	2.7 (1.4, 4.8)	11	2.7 (1.4, 4.8)
		Insecticide sprays over 7 days	0	0.0 (0.0, 52.2)	1	0.2 (0.0, 1.4)	1	0.2 (0.0, 1.4)
		Commercial repellents over 7 days	0	0.0 (0.0, 52.2)	3	0.7 (0.2, 2.2)	3	0.7 (0.2, 2.1)
		Traditional repellents over 7 days	1	20.0 (0.5, 71.6)	156	38.6 (33.8, 43.6)	157	38.4 (33.6, 43.3)
		Residual spray on interior walls in past 12 months	0	0.0 (0.0, 52.2)	46	11.4 (8.5, 14.9)	46	11.2 (8.4, 14.7)
	5–9 years	Mosquito net in the night before visit	3	100 (29.2, 100)	139	70.9 (64.0, 77.2)	142	71.4 (64.5, 77.5)
		Mosquito coils over 7 days	0	0.0 (0.0, 70.8)	3	1.5 (0.3, 4.4)	3	1.5 (0.3, 4.3)
		Insecticide sprays over 7 days	0	0.0 (0.0, 70.8)	1	0.5 (0.0, 2.8)	1	0.5 (0.0, 2.8)
		Commercial repellents over 7 days	0	0.0 (0.0, 70.8)	3	1.5 (0.3, 4.4)	3	1.5 (0.3, 4.3)
		Traditional repellents over 7 days	2	66.7 (9.4, 99.2)	68	34.7 (28.1, 41.8)	70	35.2 (28.6, 42.2)
		Residual spray on interior walls in past 12 months	0	0.0 (0.0, 70.8)	20	10.2 (6.3, 15.3)	20	10.1 (6.2, 15.1)

*Infected children* children infected with *P. falciparum* parasitaemia, *Non-infected children* children not infected with *P. falciparum* parasitaemia, *N* total number of participants, *n* number of children in a given category, % percentage of children in a given category, *95*% *CI* 95% confidence interval, *M* months, *Y* years

In Burkina Faso, the secondmost common malaria control measure was the use of mosquito coils, with a larger extent in Nouna (8.4% of children) compared to Saponé (0.8% of children). In Senegal, the secondmost common malaria control measure was the use of traditional repellents, which were not used in Burkina Faso, and the highest number of children using traditional repellents was registered in Niakhar (37.3% of children).

Exploratory analyses in Burkina Faso suggested that the use of any malaria treatment or other medication in the past 14 days at both the Nouna and Saponé sites, and the use of insecticide sprays at the Nouna site had a potential impact on the occurrence of *P. falciparum* parasitaemia (Table 4). Concerning the other factors, rural vs urban localization, natural vs cement floor, open vs closed drinking water source, and availability of electricity in the house in Nouna, and age and iron vs grass/palm roof in Saponé were also shown to have a potential impact on the occurrence of *P. falciparum* parasitaemia (Table 4). In the Senegalese centres, the 95% of all unadjusted ORs included the value ‘1’ for the assessed malaria control measures and risk factors due to the low number of infected children.

**Table 4:** Effect of malaria control measures and other factors on the proportion of occurrence of *Plasmodium falciparum* parasitaemia for the two sites in Burkina Faso

Site	Characteristic	Category	Unadjusted OR (95% CI)
Nouna	Malaria treatment in past 14 days	Yes vs no	0.323 (0.215, 0.484)
	Other medication in the past 14 days	Yes vs no	0.563 (0.403, 0.788)
	Use of insecticide sprays over 7 days	Yes vs missing/no	0.456 (0.225, 0.924)
	Age	Continuous	1.159 (1.086, 1.236)
	Localization	Rural vs urban area	2.635 (1.876, 3.701)
	Type of location	Town vs countryside	0.386 (0.275, 0.542)
	Main house construction material: floor	Natural vs cement	1.666 (1.191, 2.329)
	Main source of drinking water	Open vs closed	0.712 (0.510, 0.993)
	Presence of electricity	Yes vs no	0.401 (0.274, 0.588)
Saponé	Malaria treatment in past 14 days	Yes vs no	0.143 (0.066, 0.308)
	Other medication in the past 14 days	Yes vs no	0.300 (0.171, 0.529)
	Age	Continuous	1.329 (1.242, 1.423)
	Main house construction material: roof	Iron sheet vs grass/palm	0.631 (0.440, 0.906)

*OR* odds ratio, *95*% *CI* 95% confidence interval

## Discussion

Although efforts to control malaria infections have expanded considerably over the last years, this disease remains a major cause of morbidity and mortality in many sub-Saharan Western African countries [1, 20]. In this study, a much higher PP of *P. falciparum* was observed during the peak of malaria transmission in the two centres in Burkina Faso, where malaria endemicity is moderate to high, than in the two Senegalese centres, where malaria endemicity is low. A lower proportion of children with gametocytes, who constitute a parasite reservoir responsible for malaria transmission, was also observed in Senegal [21].

In Senegal, the prevalence of *P. falciparum* ranged between 0.5 and 1.5% across both age groups, and was the lowest at the Keur Socé site. The low prevalence observed in Keur Socé was in line with previous studies conducted in 2010 in the same area in children younger than 10 years (0.3% [95% CI 0.06–0.8]) [18], and in children younger than 5 years (1.5% [95% CI 0.7–2.6]) [22]. In Burkina Faso, 41.9–75.4% of children across both age groups and both sites were infected with *P. falciparum*. These results were in line with a previous study suggesting that the Saponé district is a stable malaria transmission area, where the malaria burden remained significant despite the introduction of effective medications and control measures [23]. However, these findings did not confirm the decline in malaria transmission previously observed in children and adults in the region of Nouna, where PP over the rainy season decreased from 78.9 in 2000 to 58.4% in 2009, 55.9% in 2010 and 49.3% in 2011 [15]. This discrepancy may be explained by between-study differences in terms of recruitment period, as participants were enrolled in October in the previous study and mainly in November in this study, and age, as this study was limited to children and the other study recruited participants from all age groups.

In this study, the prevalence of *P. falciparum* was lower in infants than in older children, which is in line with previous observations [20]. Potential explanations for the low prevalence in infants include the protection by maternal antibodies, and the fact that infants are less attractive to mosquitoes [20, 24, 25] and spend less time outdoors after sunset.

Infections with other *Plasmodium* species were scarce, with no infection with *P. vivax* and only a few cases with *P. malariae* and *P. ovale*, which is in line with other studies in Burkina Faso [15, 26], and with the World Malaria Report of WHO [2].

In Burkina Faso, malaria control policies include free distribution of ITNs, intermittent preventive treatment for pregnant women, treatment of malaria cases with ACT, and seasonal malaria chemoprevention [27]. In Senegal, malaria control measures recommended by WHO, including clinical management of malaria cases using RDTs and ACT, universal coverage of LLINs, IRS and intermittent preventive treatment, have been implemented since 2005, resulting in a decreased malaria burden [16]. These results suggest that most recommended malaria control measures were actually used at the study site level, except the distribution of ITNs which were not free of charge in Nouna and not included in the policies in Saponé, and the distribution of LLINs which was limited to children younger than 5 years and pregnant women in Niakhar. The only recommended malaria control measures evaluated at individual level were the use of mosquito bed nets and IRS. Mosquito nets were frequently used in Burkina Faso (88.6–97.0%) and Senegal (64.7–80.2%), and the majority were new and impregnated. In Burkina Faso, PP was higher than expected given the strong bed net coverage and the national recommendations for usage of malaria treatment. The similar proportion of infected and uninfected children who slept under a bed net the night before the visit and the absence of observed differences in terms of parasite density, were other unexpected findings. However, since the vast majority of children slept under a bed net, the power to detect a significant difference was low. Moreover, the proportion of children sleeping under a bed net and the number of new and impregnated bed nets might have been overestimated because bed nets were not inspected and their presence was not confirmed by study staff. Similar results were obtained in a previous study showing no effect of the ITN campaign in 2010 on the proportion of young children with *P. falciparum* parasitaemia after the rainy season in the Nouna area [28]. In Senegal, the low use of IRS was unexpected since it is recommended by the national guidelines. Besides the recommended control measures, the use of additional malaria control measures was also evaluated. In both countries, only a small number of children used mosquito coils or insecticide sprays. Traditional repellents (plant-based methods or petroleum oils [29]) were the secondmost common malaria control measure in Senegal, but these were not used in Burkina Faso.

In Burkina Faso, exploratory analyses suggested that malaria control measures having an impact on the *P. falciparum* infection status included the use of malaria treatment or other medications in the past 14 days and the use of insecticide sprays. Other factors influencing malaria occurrence included age, the main house construction material (natural floor and grass/palm roof), lack of electricity in the house, and rural residence.

The main strengths of this cross-sectional study are the indicators of the high quality of data collection, as no child was excluded from the total cohort, and the low number of protocol deviations, which were all considered as minor. The limitations included the low number of sites per country and the small sample size per site. Moreover, the results from a single cross-sectional survey should be interpreted cautiously because they do not take annual fluctuations of malaria transmission into account and they may not accurately represent the average MTI in endemic areas. The PP in Saponé could have been slightly underestimated since the peak in malaria transmission occurs in September in this area [16]. Another limitation was the potential underestimation of the PP when microscopy is used alone, especially in Senegal where malaria transmission is reduced and individuals may carry sub-microscopic malaria parasites [18].

The estimation of PP could be improved if microscopy results were confirmed by molecular detection techniques. Polymerase chain reactions (PCR) have a higher sensitivity than microscopy, but this technique is not frequently used in routine clinical practice due to its high cost. An estimate of PCR prevalence could be obtained by using a tool that has been developed to assess the relationship between microscopy and PCR measures [30]. Finally, the potential differences observed in this study and the evaluation of the impact of the various factors on the *P. falciparum* infection status were based on exploratory analyses, which should be interpreted with caution because there was no adjustment for multiplicity and no analysis on multi-collinearity was done before the regression.

## Conclusions

This study showed that the prevalence of *P. falciparum* was much higher in the two sites in Burkina Faso with moderate-to-high malaria endemicity compared with the two sites in Senegal with low malaria endemicity. Mosquito nets were frequently used in both countries, but additional malaria control measures evaluated at individual level were uncommon. Exploratory analyses suggested that control measures having an impact on the *P. falciparum* infection status included the use of malaria treatment, other medications and insecticide sprays. In the centres with moderate-to-high malaria endemicity, age, main house construction material and lack of electricity in the house were also associated with an increased malaria occurrence. The high PP observed in the two sites in Burkina Faso suggests that new malaria control measures are needed in these areas.

## Abbreviations

ACT: artemisinin-based combination therapy
CI: confidence interval
HDSS: Health and Demographic Surveillance Systems
IRS: indoor residual spraying
ITN: insecticide-treated net
JTEG: Joint Technical Expert Group
LLIN: long-lasting insecticidal net
MTI: intensity of malaria transmission
OR: odds ratio
PCR: polymerase chain reaction
PP: parasite prevalence
RDT: rapid diagnostis test
SAS: statistical analysis systems
SD: standard deviation
WHO: World Health Organization
